# The Association Between Increased Levels of Patient Engagement With an Internet Support Group and Improved Mental Health Outcomes at 6-Month Follow-Up: Post-Hoc Analyses From a Randomized Controlled Trial

**DOI:** 10.2196/10402

**Published:** 2018-07-17

**Authors:** Emily M Geramita, Bea Herbeck Belnap, Kaleab Z Abebe, Scott D Rothenberger, Armando J Rotondi, Bruce L Rollman

**Affiliations:** ^1^ Division of General Internal Medicine University of Pittsburgh Medical Center Pittsburgh, PA United States; ^2^ Department of Psychosomatic Medicine and Psychotherapy University of Göttingen Medical Center Göttingen Germany; ^3^ Center for Behavioral Health and Smart Technology University of Pittsburgh School of Medicine Pittsburgh, PA United States; ^4^ Veterans Affairs Pittsburgh Healthcare System Pittsburgh, PA United States

**Keywords:** internet support group, patient engagement, anxiety, depression

## Abstract

**Background:**

We recently reported that depressed and anxious primary care patients randomized to a moderated internet support group (ISG) plus computerized cognitive behavioral therapy (cCBT) did not experience improvements in depression and anxiety over cCBT alone at 6-month follow-up.

**Objective:**

The 1% rule posits that 1% of participants in online communities generate approximately 90% of new user-created content. The aims of this study were to apply the 1% rule to categorize patient engagement with the ISG and identify whether any patient subgroups benefitted from ISG use.

**Methods:**

We categorized the 302 patients randomized to the ISG as: superusers (3/302, 1.0%), top contributors (30/302, 9.9%), contributors (108/302, 35.8%), observers (87/302, 28.8%) and those who never logged in (74/302, 24.5%). We then applied linear mixed models to examine associations between engagement and 6-month changes in health-related quality of life (HRQoL; Short Form Health Survey Mental Health Component, SF-12 MCS) and depression and anxiety symptoms (Patient-Reported Outcomes Measurement Information System, PROMIS).

**Results:**

At baseline, participant mean age was 42.6 years, 81.1% (245/302) were female, and mean Patient Health Questionnaire (PHQ-9), Generalized Anxiety Disorder scale (GAD-7), and SF-12 MCS scores were 13.4, 12.6, and 31.7, respectively. Of the 75.5% (228/302) who logged in, 61.8 % (141/228) created ≥1 post (median 1, interquartile range, IQR 0-5); superusers created 42.3 % (630/1488) of posts (median 246, IQR 78-306), top contributors created 34.6% (515/1488; median 11, IQR 10-18), and contributors created 23.1 % (343/1488; median 3, IQR 1-5). Compared to participants who never logged in, the combined superuser + top contributor subgroup (n=33) reported 6-month improvements in anxiety (PROMIS: –11.6 vs –7.8; *P*=.04) and HRQoL (SF-12 MCS: 16.1 vs 10.1; *P*=.01) but not in depression. No other subgroup reported significant symptom improvements.

**Conclusions:**

Patient engagement with the ISG was more broadly distributed than predicted by the 1% rule. The 11% of participants with the highest engagement levels reported significant improvements in anxiety and HRQoL.

**Trial Registration:**

ClinicalTrials.gov NCT01482806; https://clinicaltrials.gov/ct2/show/NCT01482806 (Archived by WebCite at http://www.webcitation.org/708Bjlge9).

## Introduction

### Background

Internet support groups (ISGs) are specialized social media websites that connect individuals with common health conditions and provide a forum for peers to exchange information, resources, and support [1,2]. While ISGs for mental health conditions have become increasingly common [3], randomized trials [4,5] and systematic reviews [6-8] find they have mixed benefits for reducing psychologic distress. In a recent randomized controlled trial, we reported that providing depressed and anxious primary care patients with access to a moderated ISG in addition to a computerized cognitive behavioral therapy (cCBT) program provided no additional intent-to-treat benefit in patients’ health-related quality of life (HRQoL) or mood and anxiety symptoms over the cCBT program alone at 6-month follow-up, although cCBT was more effective than primary care physicians’ (PCPs) usual care [9] (NCT01482806). These null findings raise questions about whether any subgroups of ISG members may have benefitted differentially from the ISG based on their level of engagement.

One approach to classify engagement with an online community is the 1% rule [10,11]. Adapted from the digital marketing literature, the 1% rule posits that 1% of online community members (superusers) create approximately 90% of user-generated content, approximately 10% of members (contributors) create less than 10% of the remaining content, and 90% of members (observers) rarely contribute but mainly observe activity. A recent observational study replicated the 1% rule in 4 large ISGs for individuals with addiction and mood disorders [12] and found that participants’ demographic and disease-specific characteristics were not associated with their level of engagement with these online communities [13].

### Goal of This Study

Very little work has been done to investigate the relationship between level of ISG engagement and clinical outcomes for treating depression and anxiety or any other mental health condition in primary care [5,14]. Therefore, to classify the patients randomly assigned to our trial’s ISG arm by their level of engagement, we applied the 1% rule based on the number of posts they created on the ISG. We then conducted post hoc analyses to compare these engagement level subgroups with patients randomly assigned to the ISG who never logged in to examine whether any patient subgroup benefitted from participating in our online community.

## Methods

### Study Setting, Patient Eligibility, and Randomization and Experimental Conditions

The protocol for the Online Treatment for Mood and Anxiety Disorders Trial was approved by the University of Pittsburgh’s Institutional Review Board and detailed in the trial’s primary outcomes report [9]. Briefly, PCPs in 26 southwestern Pennsylvania practices referred patients with a Generalized Anxiety Disorder scale (GAD-7) [15] or Patient Health Questionnaire (PHQ-9) [16] score ≥10, indicating moderately severe anxiety or depression symptoms, between August 2012 and September 2014. We randomized 704 protocol-eligible participants to either (1) care manager–guided access to the 8-session “Beating the Blues” cCBT program designed to provide users with basic CBT skills [17] (cCBT-only; 301/704), (2) cCBT plus additional access to our password-protected and moderated ISG (ISG+cCBT; 302/704), or (3) their PCP’s usual care (101/704). All study arms had similar baseline sociodemographic and clinical characteristics [9]. Analyses in this report focus solely on the 302 participants assigned to the ISG+cCBT arm.

### Care Manager Support

Following randomization, the care manager exclusively assigned to the ISG+cCBT arm contacted each participant via telephone to provide basic psychoeducation and encourage them to start the cCBT program and log in to the ISG. Later, he contacted participants (we define these as care manager contacts) via email, text, and telephone to promote adherence with the cCBT program and treatment recommendations, including suggestions to access various resources on the ISG. The care manager presented each participant’s progress to the study PCP, psychiatrist, and psychologist at a weekly case review meeting [18].

### Internet Support Group

**Figure 1:**
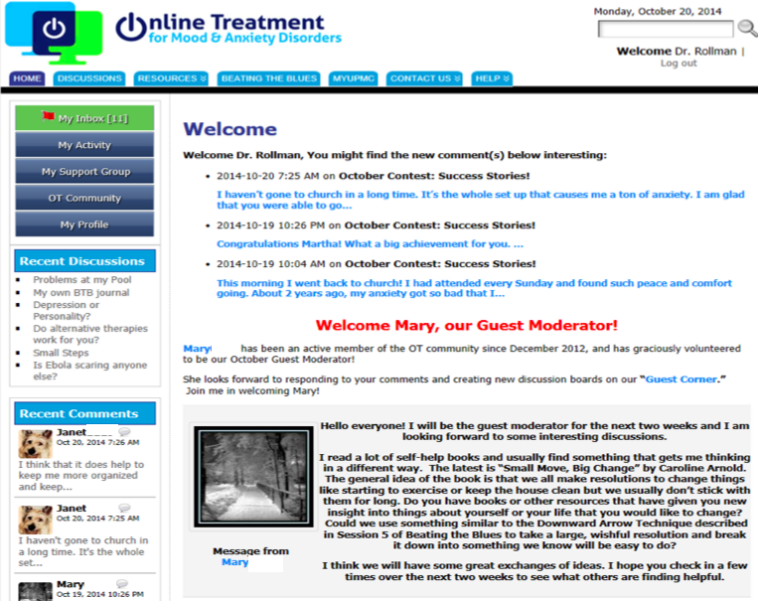
Screenshot of our internet support group homepage (ottrial.pitt.edu).

We used WordPress (Automattic Inc) software to create our password-protected ISG that was accessible via computer or smartphone (Figure 1). The ISG featured moderated discussion boards created by the care manager–ISG moderator and study participants. The study team used an iterative process to decide on the initial discussion board topics, with a focus on common challenges faced by patients with depression and anxiety (eg, managing symptoms, discussing mental health issues with friends, common triggers). The ISG also curated links to external resources including local $4 generic pharmacy programs; find-a-therapist; crisis hotlines; brief YouTube videos on insomnia, nutrition, exercise, and other topics; our electronic medical record system’s patient portal; and the cCBT program (Multimedia Appendix 1).

To preserve confidentiality, we assigned members usernames and regularly reminded them not to post any self-identifying information or photographs. Additionally, a study investigator logged in to the ISG daily to review new posts for suicidal thoughts and other potentially inappropriate content. Participants were also able to flag comments for review by the ISG moderator and possible removal.

### Engagement with the Internet Support Group

We provided participants with password-protected access to the ISG approximately 3 months after the start of subject enrollment once the first 25 patients were randomized to the ISG arm to promote user-generated activity. Afterward, we provided participants with ISG access shortly after randomization.

Participants created content on the ISG discussion boards by either initiating a new discussion thread or commenting on an ongoing thread (posts). On most weeks, the care manager–ISG moderator also initiated new discussion threads on such topics as coping with mental health symptoms, talking about depression and anxiety with friends, stressors (eg, holidays, work-life balance), and lifestyle challenges (eg, healthy diet, losing weight, exercise).

Although we encouraged participants to log in and post on the ISG throughout their 6-month intervention phase, we did not require them to do so. Still, we took several measures to encourage participants to log in and post by featuring status indicators on their profiles and posts (eg, stars and likes), emailing notifications of new ISG activities and posts, highlighting new posts on their homepage based on their past ISG activity, inviting participants to serve as guest moderators, and holding various contests that promoted logging in and posting.

### Assessments

Following confirmation of protocol eligibility and consent, a study assessor collected sociodemographic and clinical information from our study practices’ electronic medical record system and from the participant, ascertained self-identified race, and administered the Primary Care Evaluation of Mental Disorders (PRIME-MD) Anxiety and Mood Modules to establish a psychiatric diagnosis [19], the 12-Item Short Form Health Survey Mental Components Score (SF-12 MCS) to measure HRQoL [20], and the Patient-Reported Outcomes Measurement Information System (PROMIS) Depression and Anxiety short forms to measure depression and anxiety symptom levels [21]. Later, an assessor who was blinded to participants’ randomization assignment telephoned participants to readminister the PROMIS and SF-12 MCS at 3 months and at the 6-month primary outcome time point.

We obtained counts of unique patient log-ins and posts from the logs of the server that hosted the ISG. We defined a post as an entry that initiated a new discussion thread or added an entry to an existing discussion thread, and we summarized the number of posts each participant made to arrive at a total.

### Classification of Internet Support Group Engagement

Using the 1% rule as our starting point, we classified participants into subgroups by level of engagement as measured by the total number of posts each created during the first 6 months after randomization (top 1% of posters, next 9%, and remaining 90%) [10,11]. Given our interest in identifying the gradient of participant engagement, we further classified participants into the following subgroups: superusers (top 1%), top contributors (next 9%), contributors (made at least 1 post), observers (logged in at least once but never posted), and those who never logged in. Since several participants between the 9th and 11th percentiles made the same number of posts, we reclassified our top contributors as the next highest 10% of posters after superusers, rather than the next 9%.

### Statistical Analysis

We calculated the baseline sociodemographic and clinical characteristics across the 5 ISG engagement groups using percentages, means and standard deviations, and medians and interquartile ranges (IQR), and we made group comparisons using analysis of variance and chi-square tests. As we had only 3 superusers, we grouped them with the 30 top contributors for all analyses to conduct more meaningful comparisons.

We used linear mixed models for each of the clinical outcomes (SF-12 MCS, PROMIS Depression, PROMIS Anxiety) that included fixed effects for engagement subgroup, time, group-by-time interaction, education, self-identified race, gender, and random effects for participants. We also compared the 6-month change in HRQoL and depression and anxiety symptoms between participants who were assigned to the ISG arm (ISG+cCBT) but never logged in and participants in our combined superuser + top contributor subgroup. All analyses were conducted using SAS version 9.4 (SAS Institute Inc).

## Results

### Baseline Sociodemographic and Clinical Characteristics

At baseline (Table 1), the 302 participants randomized to the ISG+cCBT arm reported moderately severe depression (PHQ-9 mean 13.4, SD 4.7) and anxiety symptoms (GAD-7 mean 12.6, SD 4.5) and low HRQoL (SF-12 MCS mean 31.7, SD 9.4). They had a mean age of 42.6 years, 81.1% (245/302) were female, and 47.7% (144/302) had at least a college education.

While each engagement subgroup was predominately female, white, and had comorbid depression and anxiety, reflecting the overall composition of our study cohort, participants who were female, white, and college educated were more likely to be in the superusers + top contributors subgroup (eg, ≥4-year college education: 70%, 23/33 superusers + top contributors vs 36%, 27/74 of the never log-ins; Table 1).

### Distribution of Engagement

Seventy-five percent of participants (228/302) logged in to the ISG at least once during their 6-month intervention phase, for a total of 2041 log-ins. Of those, the median number of log-ins per participant was 4 (IQR 2-9.5; range 1-214). Participants created 1488 posts over the 6-month intervention phase, and 61.8% (141/228) made at least 1 post (median posts per participant: 1, IQR 0-5).

As expected, the mean number of log-ins and posts differed widely across engagement subgroups (*P*<.001, Tables 2 and 3). However, the distribution of posts in our sample was less skewed than predicted by the 1% rule, with superusers making 42.3% (630/1488) of posts (median 246, IQR 78-306), top contributors 34.6% (515/1488, median 11, IQR 10-18) and contributors 23.1% (343/1488, median 3, IQR 1-5). Moreover, only 28.8% (87/302) of participants in the ISG were classified as observers (ie, they logged in to the site at least once but never posted).

### Process Measures of Care

Overall, the mean number of cCBT sessions completed was 5.5 (SD 2.7), and 35.8% (108/302) completed all 8 cCBT sessions. Across engagement subgroups, participants who created more posts also completed more cCBT sessions (*P*<.001) and had more care manager contacts (*P*<.001; Tables 2 and 3).

### Mental Health Outcomes at 6 Months

After adjusting for gender, race, and education level, all engagement subgroups reported similar improvements in symptoms at 6-month follow-up regardless of level of engagement with the ISG (Tables 4 and 5). Furthermore, compared to participants who never logged in to the ISG, the combined superusers + top contributors subgroup reported a greater improvement in HRQoL (mean ∆ SF-12 MCS: 16.1, SE 1.9 vs 10.1, SE 1.3, *P*=.01) and anxiety symptoms (mean ∆ PROMIS T-score: –11.6, SE 1.5 vs –7.8, SE 1.0, *P*=.04); we did not observe a similar improvement in depression symptoms.

**Table 1:** Baseline sociodemographic and psychiatric characteristics by engagement level.

Characteristic		Overall (n=302)	Superusers + top contributors (n=33)	Contributors (n=108)	Observers (n=87)	Never log-ins (n=74)	*P* value^a^
Age, mean (SD)		42.6 (14.4)	40.9 (13.3)	41.9 (14.4)	43.0 (14.0)	43.9 (15.5)	.72
Female, n (%)		245 (81.1)	31 (94)	88 (81.4)	63 (72)	63 (85)	.04^b^
White race, n (%)		242 (80.1)	29 (88)	94 (87.0)	65 (75)	54 (73)	.04^b^
≥4-year college degree, n (%)		144 (47.7)	23 (70)	54 (50.0)	40 (46)	27 (36)	.02
Married or living with partner, n (%)		120 (39.7)	18 (55)	42 (38.9)	36 (41)	24 (32)	.38
Employed, n (%)		204 (67.6)	22 (67)	75 (69.4)	62 (71)	45 (61)	.52
**Psychiatric^c^ diagnosis, n (%)**							.75^b^
	Major depression only	63 (21.6)	8 (24)	21 (19.4)	21 (24)	13 (18)	
	Generalized anxiety disorder only	22 (7.5)	3 (9)	5 (4.6)	7 (8)	7 (9)	
	Both depression and anxiety	207 (70.9)	22 (67)	78 (72.2)	57 (66)	50 (68)	
PHQ-9^d^, mean (SD)^e^		13.4 (4.7)	12.3 (5.5)	14.0 (4.4)	13.2 (4.6)	13.3 (4.7)	.27
GAD-7^f^, mean (SD)^e^		12.6 (4.5)	13.3 (4.8)	12.8 (4.6)	12.6 (4.7)	12.1 (3.8)	.62
PROMIS^g^ Depression T-score, mean (SD)		62.0 (6.3)	61.8 (6.7)	62.4 (6.1)	62.0 (6.5)	61.5 (6.1)	.79
PROMIS Anxiety T-score, mean (SD)		65.8 (6.2)	66.9 (6.7)	66.0 (6.5)	65.6 (5.4)	65.2 (6.2)	.57
SF-12 MCS^h^, mean (SD)		31.7 (9.4)	31.4 (8.9)	30.9 (9.2)	31.2 (8.5)	33.6 (10.9)	.25
Depression/anxiety medication use in past year, n (%)		236 (78.1)	26 (79)	87 (80.6)	68 (78)	55 (74)	.66^b^
Mental health therapist visit in past year, n (%)		59 (19.5)	10 (30)	18 (16.7)	19 (22)	12 (16)	.35

^a^*P* value represents comparison of the 4 engagement level groups.

^b^*P* value from Fisher exact test.

^c^10 participants did not meet diagnostic criteria for depression or anxiety on the Primary Care Evaluation of Mental Disorders; these participants were not included in the denominator when calculating the percentage with each diagnosis.

^d^PHQ-9: Patient Health Questionnaire.

^e^n=30 in Superusers and Top contributors group.

^f^GAD-7: Generalized Anxiety Disorder scale.

^g^PROMIS: Patient-Reported Outcomes Measurement Information System.

^h^SF-12 MCS: Short Form Health Survey Mental Components Score.

**Table 2:** Six-month internet support group log-ins, posts, and process measures by engagement level across all groups.

Characteristic		Superusers (n=3)	Top contributors (n=30)	Contributors (n=108)	Observers (n=87)	Never log-ins (n=74)
**ISG^a^ log-ins**						
	Mean (SD)	119.0 (84.3)	22.5 (16.5)	7.3 (6.6)	2.5 (2.1)	N/A^b^
	Median (IQR^c^)	90 (53-214)	18 (13-27)	5.5 (3-9)	2 (1-3)	N/A
**ISG posts**						
	Mean (SD)	210.0 (118.2)	17.2 (13.3)	3.2 (2.1)	N/A	N/A
	Median (IQR)	246 (78-306)	11 (10-18)	3 (1-5)	N/A	N/A
**cCBT^d^ sessions completed**						
	Mean (SD)	8.0 (0.0)	7.4 (1.4)	5.8 (2.6)	4.2 (3.0)	1.9 (2.7)
**Care manager contacts**						
	Mean (SD)	36.0 (11.8)	19.4 (5.6)	18.4 (6.4)	15.7 (5.0)	13.1 (4.7)

^a^ISG: internet support group.

^b^N/A: not applicable.

^c^IQR: interquartile range.

^d^cCBT: computerized cognitive behavioral therapy.

**Table 3:** Six-month internet support group log-ins, posts and process measures by engagement level with combined superusers + top contributors group.

Characteristic		Superusers + top contributors (n=33)	Contributors (n=108)	Observers (n=87)	Never log-ins (n=74)	*P* value
**ISG^a^ log-ins**						
	Mean (SD)	31.2 (38.5)	7.3 (6.6)	2.5 (2.1)	N/A^b^	<.001
	Median (IQR^c^)	20 (13, 31)	5.5 (3, 9)	2 (1, 3)	N/A	<.001^d^
**ISG posts**						
	Mean (SD)	34.7 (64.8)	3.2 (2.1)	N/A	N/A	<.001
	Median (IQR)	12 (10, 14)	3 (1, 5)	N/A	N/A	<.001^d^
**cCBT^e^ sessions completed**						
	Mean (SD)	7.4 (1.3)	5.8 (2.6)	4.2 (3.0)	1.9 (2.7)	<.001
**Care manager contacts**						
	Mean (SD)	20.9 (7.8)	18.4 (6.4)	15.7 (5.0)	13.1 (4.7)	<.001

^a^ISG: internet support group.

^b^N/A: not applicable.

^c^IQR: interquartile range.

^d^*P* value from Kruskal-Wallis test.

^e^cCBT: computerized cognitive behavioral therapy.

**Table 4:** Mental health outcomes by engagement level across all groups^a^.

Characteristic			Superusers + top contributors (n=33)	Contributors (n=108)	Observers (n=87)	Never log-ins (n=74)	*P* value
**SF-12 MCS^b^, estimated mean (SE)^c^**							
	Baseline		31.2 (1.9)	30.9 (1.2)	31.2 (1.2)	33.8 (1.3)	N/A^d^
	6 months		47.2 (2.0)	42.5 (1.2)	43.8 (1.2)	44.0 (1.4)	N/A
	**Δ 6 months**		16.1 (1.9)	11.7 (1.1)	12.6 (1.2)	10.1 (1.3)	.08
		Superusers + top contributors vs never log-in	16.1 (1.9)	N/A	N/A	10.1 (1.3)	.01
**PROMIS^e^** **Depression T-score, estimated mean (SE)^f^**							
	Baseline		62.0 (1.5)	62.3 (0.9)	61.8 (0.9)	61.1 (1.0)	N/A
	6 months		51.7 (1.5)	54.1 (0.9)	53.2 (0.9)	53.5 (1.1)	N/A
	**Δ 6 months**		–10.3 (1.3)	–8.2 (0.8)	–8.6 (0.8)	–7.6 (0.9)	.39
		Superusers + top contributors vs never log-in	-10.3 (1.3)	N/A	N/A	-7.6 (0.9)	.09
**PROMIS Anxiety T-score, estimated mean (SE)^g^**							
	Baseline		67.2 (1.5)	66.1 (0.9)	65.6 (0.9)	65.2 (1.0)	N/A
	6 months		55.7 (1.5)	57.6 (0.9)	56.3 (0.9)	57.4 (1.1)	N/A
	**Δ 6 months**		–11.6 (1.5)	–8.5 (0.8)	–9.4 (0.9)	–7.8 (1.0)	.19
		Superusers + top contributors vs never log-in	-11.6 (1.5)	N/A	N/A	-7.8 (1.0)	.04

^a^All models are adjusted for gender, race, and education; n=259 (25 participants were missed at the 6-month assessment, and 9 participants withdrew from the study).

^b^SF-12 MCS: Short Form Health Survey Mental Components Score.

^c^Range 0-100; higher scores indicate better health-related quality of life.

^d^N/A: not applicable.

^e^PROMIS: Patient-Reported Outcomes Measurement Information System.

^f^T-score range 37.1-81.1; lower scores indicate less severe symptoms.

^g^T-score range 36.3-82.7; lower scores indicate less severe symptoms.

**Table 5:** Mental health outcomes by internet support group (ISG) log-in status^a^.

Characteristic		Logged in to ISG ≥1 time^b^ (n=228)	Never logged in to ISG (n=78)	*P* value
**SF-12 MCS^c^, estimated mean (SE)^d^**				
	Δ 6 months, log-in vs never log-in	12.6 (0.7)	10.1 (1.3)	.11
**PROMIS^e^Depression T-score, estimated mean (SE)^f^**				
	Δ 6 months, log-in vs never log-in	-8.7 (0.5)	-7.6 (0.9)	.31
**PROMIS Anxiety T-score, estimated mean (SE)^g^**				
	Δ 6 months, log-in vs never log-in	-9.3 (0.6)	-7.8 (1.0)	.20

^a^All models are adjusted for gender, race, and education; n=259 (25 participants were missed at the 6-month assessment, and 9 participants withdrew from the study).

^b^Includes superusers, top contributors, contributors, and observers.

^c^SF-12 MCS: Short Form Health Survey Mental Components Score.

^d^Range 0-100; higher scores indicate better health-related quality of life.

^e^PROMIS: Patient-Reported Outcomes Measurement Information System.

^f^T-score range 37.1-81.1; lower scores indicate less severe symptoms.

^g^T-score range 36.3-82.7; lower scores indicate less severe symptoms.

## Discussion

### Principal Findings

To the best of our knowledge, this is the first report to demonstrate that high levels of patient engagement with a moderated ISG, compared to no engagement with the ISG, are associated with improved anxiety symptoms and HRQoL in primary care. Our findings also provide further empirical evidence to support the participation inequality suggested by the 1% rule, although we observed a broader distribution of posting than posited by the 1% rule.

Our work confirms that depressed and anxious primary care patients are willing to engage in an ISG even when not required by study protocol to do so. Indeed, the sizable majority of our study subjects logged in to the ISG at least once, which is consistent with log-in rates reported in other studies of ISGs for depression [4,22]. Furthermore, among those who logged in to the ISG, participation inequality was less extreme than expected based on the 1% rule, as our top 1% and 10% of posters together generated 78% of all user-created content on our site, not 99% as the 1% rule predicts. Still, challenges remain in developing even more equitably engaged online communities to improve health and HRQoL.

Prior work on the impact of ISG engagement has been limited largely to comparing psychosocial outcomes between posters (defined as individuals who made at least 1 post) and observers in ISGs for women with breast cancer [23,24]. Findings from this work were mixed: while a moderate sized cross-sectional study showed more benefits in perceived social support in posters than observers [23], a large prospective study showed higher perceived functional well-being and fewer mood symptoms in observers than posters at 3-month follow-up [24]. To our knowledge, the only other study to explore the impact of engagement on mood symptoms in ISGs for mental health measured engagement by time spent on the ISG, showing that members who spent more than 5 hours on the ISG over a 2-week period were more likely to have resolution of depression at 6 months than members who spent less time [4].

Our finding that the participants who were highly engaged with the ISG reported improved anxiety symptoms and HRQoL at 6 months compared to individuals who never logged in identifies a subgroup that may benefit from participating in an ISG. Interestingly, this group did not report similar benefit for depression symptoms compared to the group that never logged in. On average, this highly engaged subgroup posted 5.8 times per month, which averaged approximately 1 post per log-in. Demographically, this subgroup had higher proportions of participants who were female, white, and college-educated than the group that never logged in, but both groups had similar levels of baseline depression and anxiety. This finding offers encouragement about the potential for ISGs to improve clinical outcomes in individuals who engage highly with an ISG. Still, more work is needed to confirm our findings in a randomized trial and identify the critical threshold of engagement needed to demonstrate clinically meaningful improvements in health.

Our work motivates further study into how to most accurately measure engagement with an ISG. We quantified engagement using the relatively simple metric of number of posts, and we assigned each post an equivalent weight. However, other quantitative metrics such as time spent on the ISG and number of pages viewed may offer a different perspective. Moreover, qualitative metrics that analyze post content may also be an important dimension of engagement, particularly considering evidence from breast cancer ISGs suggesting that a subset of members derive psychological benefit from creating posts that provide “insightful disclosure” [25].

### Limitations

Our study has several limitations. First, our finding that high levels of engagement improved clinical outcomes reflects a post hoc analysis that we undertook to identify a subgroup that may have benefitted from the ISG. Second, the limited size of the ISG precluded further subgroup analyses and required us to combine the superuser and top contributor groups for all outcome analyses. Third, we quantified engagement using a simple measure of post counts, and we used this measure to stratify the sample into engagement levels based on the 1% rule rather than statistical methods that avoid specifying an a priori hypothesis about engagement distribution. Fourth, we were not able to include a content analysis of posts or examine the impact of post content on outcomes. Finally, since all participants had access to the cCBT program, we cannot exclude that the overall improvements we observed could be attributed to the cCBT program given its demonstrated efficacy [9,26].

### Implications

Our work shows that primary care patient engagement in an online community for depression and anxiety may contribute to improved mental health at 6 months after enrollment but only at the highest levels of engagement. We strongly encourage researchers, clinicians, and health care delivery systems considering deployment of a similar ISG to first develop plans to encourage and sustain high and broad levels of user engagement. Future work is needed to (1) confirm our findings with mental health and other conditions, (2) establish the threshold of patient engagement required to benefit from an ISG, and (3) perform content and other qualitative analyses of discussion board posts to explore the influence of this content with patient engagement and other outcomes of interest.

### Conclusions

In summary, we demonstrated that patient engagement with our moderated ISG for depressed and anxious primary care patients generally approximates the 1% rule. Although we observed broader engagement levels with the ISG than predicted by the 1% rule, only ISG members who engaged at the highest levels of engagement reported measurable improvement in symptoms and quality of life at 6-month follow-up.

## Appendix

Multimedia Appendix 1 Screenshots of our internet support group.

## Abbreviations

cCBT: computerized cognitive behavioral therapy
GAD-7: Generalized Anxiety Disorder scale
HRQoL: health-related quality of life
IQR: interquartile range
ISG: internet support group
PCP: primary care physician
PHQ-9: Patient Health Questionnaire
PRIME-MD: Primary Care Evaluation of Mental Disorders
PROMIS: Patient-Reported Outcomes Measurement Information System
SF-12 MCS: Short Form Health Survey Mental Components Score
