# Supplementary material for: The Association Between Increased Levels of Patient Engagement With an Internet Support Group and Improved Mental Health Outcomes at 6-Month Follow-Up: Post-Hoc Analyses From a Randomized Controlled Trial
Source: J Med Internet Res. 2018 Jul 17;20(7):e10402. doi: 10.2196/10402 (PMC6068384; doi:10.2196/10402)
Supplement: Multimedia Appendix 1 [file jmir_v20i7e10402_app1.pptx]

## Slide 1
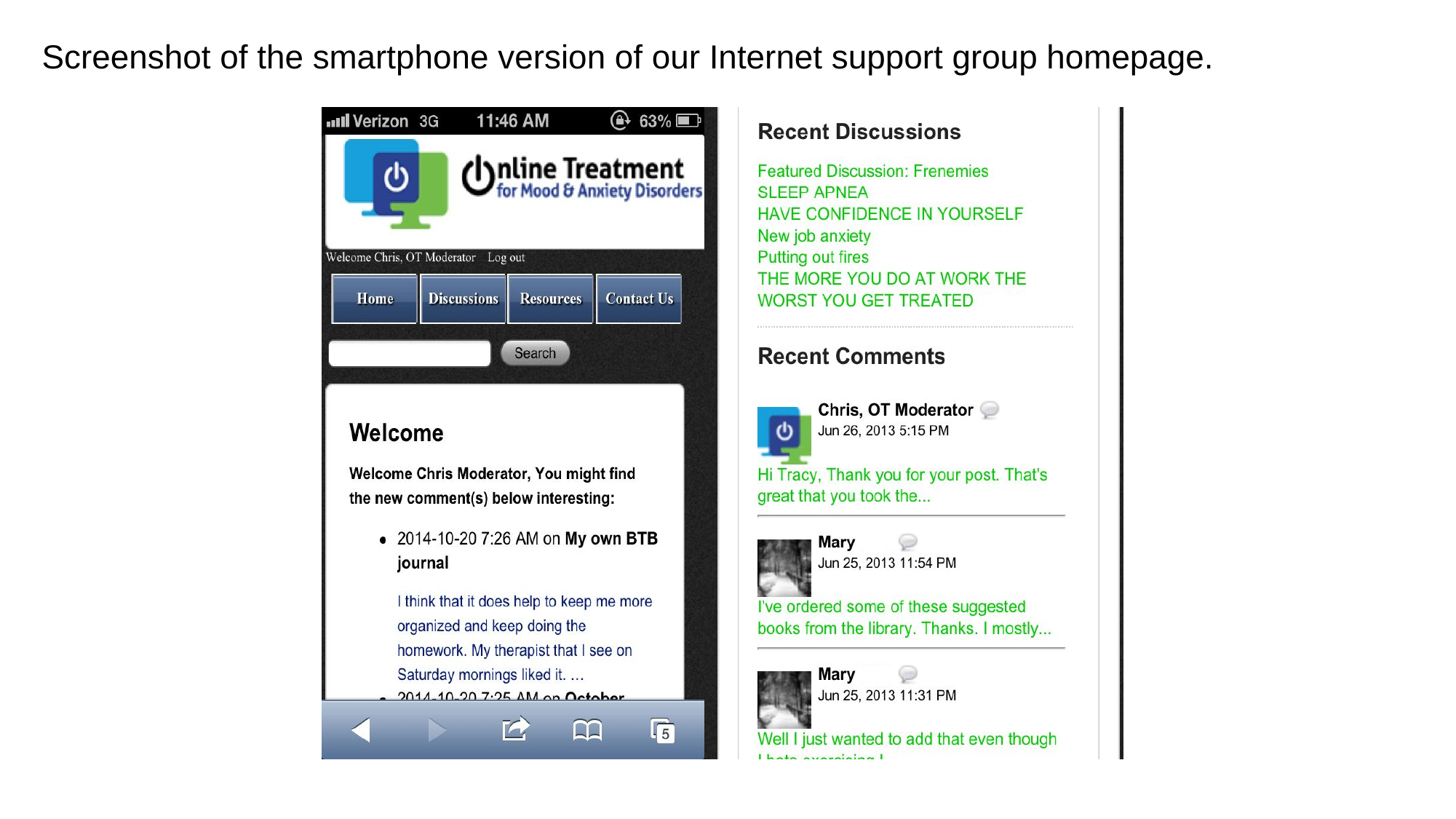

Screenshot of the smartphone version of our Internet support group homepage.

## Slide 2
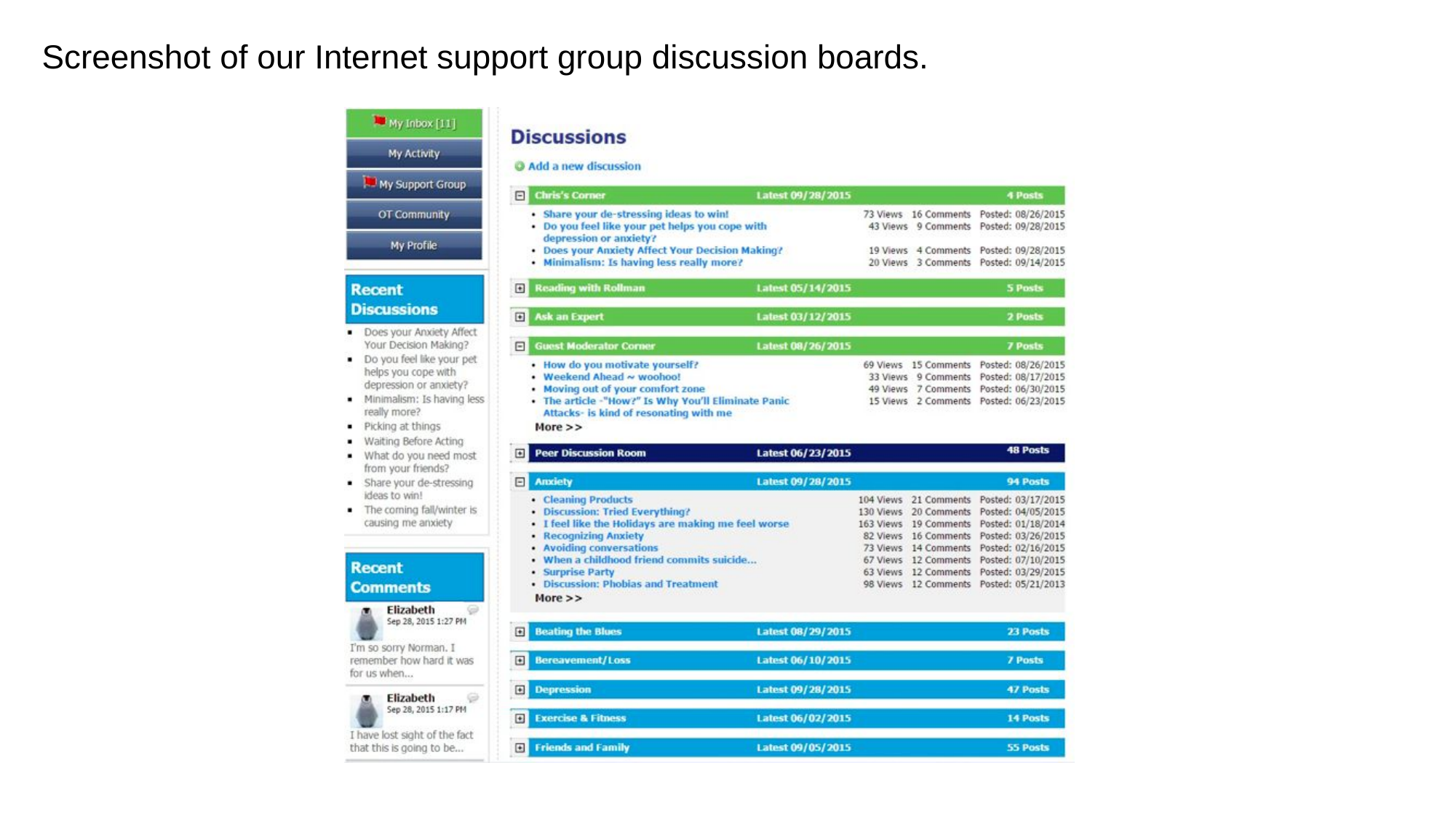

Screenshot of our Internet support group discussion boards.

## Slide 3
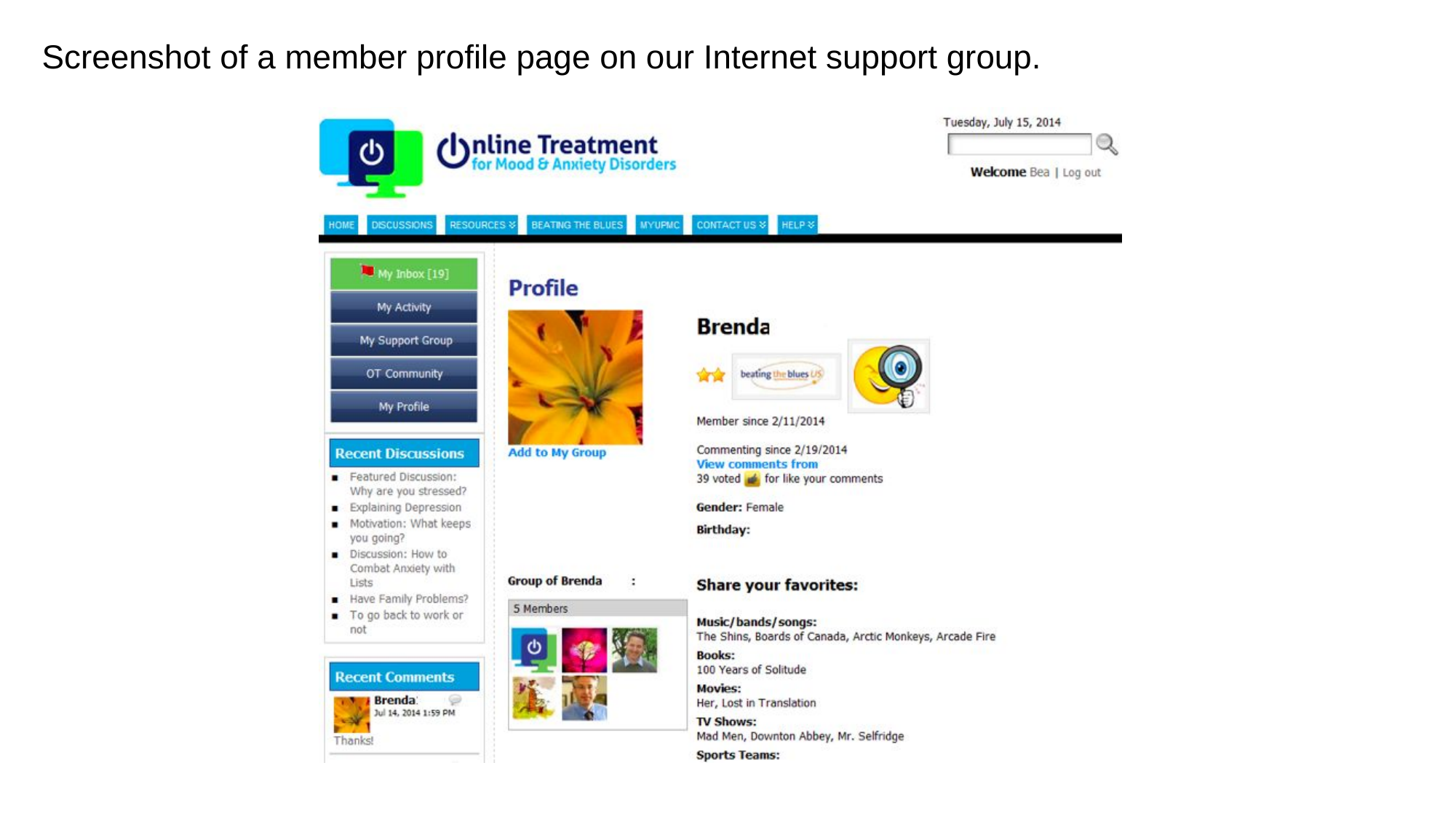

Screenshot of a member profile page on our Internet support group.
